# Cyclothymic and anxious affective temperament in perinatal depression: findings from an exploratory cross-sectional study

**DOI:** 10.3389/fpsyt.2026.1790781

**Published:** 2026-04-23

**Authors:** Laura Orsolini, Giulio Longo, Eleonora Manfredi, Federica Tassi, Rosa Volgare, Cristina Romaldi, Giulia Francesconi, Umberto Volpe

**Affiliations:** Unit of Clinical Psychiatry, Department of Clinical Neurosciences/DIMSC, Polytechnic University of Marche, Ancona, Italy

**Keywords:** affective temperaments, depression, perinatal depression, pregnancy, women’s mental health

## Abstract

**Introduction:**

The perinatal period represents a vulnerable period in which women may experience high psychic distress due to psychological, biological and social changes. The prevalence of perinatal depression (PND) is estimated around 15%-20% during pregnancy and 16%-18% after childbirth. Although several risk factors have been investigated in the PND development, few studies explored the role of affective temperaments, well known to exert a role in any mood disorders. The aim of our study was to explore which is the most represented affective temperamental profile in PND as well as which is its role in the development and severity of depressive symptoms during perinatal period.

**Methods:**

All pregnant women admitted at the Perinatal Mental Health Outpatient Service, Unit of Clinical Psychiatry, University Hospital of Marche, Polytechnic University of Marche, Ancona, Italy, between April 2021 and July 2025, were screened for PND through Edinburgh Postnatal Depression Scale (EPDS) and a semi-structured clinical interview (SCID-5-CV). Temperament Evaluation of Memphis, Pisa, Paris and San Diego (TEMPS-M) was administered to all pregnant women.

**Results:**

The PND prevalence was 33.1%. PND was significantly associated with higher cyclothymic (B = 0.356, p = 0.001) and anxious TEMPS-M scores (B = 0.247, p = 0.026) and a positive psychiatric history (B = 5.245, p < 0.001) (R = 0.6, R2 = 0.36, F(3,129) = 24.189, p < 0.001). Logistic regression indicated that cyclothymic (Exp(B)=1.118, p=0.008), hyperthymic (Exp(B)=0.911, p=0.049), anxious temperaments (Exp(B)=1.109, p=0.029), presence of medical comorbidities (Exp(B)=0.224, p=0.003) and psychiatric history (Exp(B)=5.144, p=0.001) were independent predictors of PND.

**Discussion:**

Affective temperaments, particularly cyclothymic and anxious profiles, and prior psychiatric history are predictors of perinatal depression. Incorporating temperament assessment alongside standard screening tools such as the EPDS may improve early identification of women at risk, supporting tailored preventive and therapeutic strategies.

## 1 Introduction

Pregnancy is a potential vulnerable period in a woman’s life, characterized by profound physical, psychological as well as emotional changes. During the whole period, hormonal and psychological alterations may lead to a complex emotional turmoil ranging from euphoria, dysphoria, anticipatory anxiety, phobia, stress and depression (1). The perinatal period may overall determine a significant impact on affective spectrum disorders, particularly in those mental conditions already existing in the pre-conception period (2). Indeed, it may occur a *de novo* onset and/or a recrudescence of an affective spectrum disorder, both during the pregnancy and/or in the postpartum period, particularly with the clinical manifestation of a condition afferent to a major depressive disorder (MDD), i.e. perinatal depression (PND). PND is the term used to refer to any non-psychotic depressive episode occurring during pregnancy and/or in the postpartum up to one year of childbirth (3–5). The clinical picture of PND is similar to MDD diagnostic criteria, including depressed mood, lack of pleasure or interest, changes in appetite, sleep disorders, agitation, restlessness/slowness, reduced energy, easy fatigue and exhaustion, feelings of worthlessness, guilt, brain fog, recurring thoughts of life not being worth living, suicidal thoughts (6, 7).

Recent systematic reviews estimated a PND prevalence of around 15%-20% during pregnancy and 16%-18% after childbirth, with higher rates observed in low- and middle-income countries (2, 5, 8). In Italy, it has been documented a high epidemiological variability among those studies specifically investigating PND prevalence in clinical samples, mainly due to the clinical small sample size (9–12). Indeed, a recent systematic review estimated an antepartum PND rate of 20.2% and a postpartum PND rate of 27.5% (13). The consequences of PND may extend beyond the mother’s mental health, negatively impacting mothers’ quality of life and intimate relationships, influencing birth outcomes, outcomes related to subsequent breastfeeding, and the long-term effects on children’s cognitive and emotional development (14). Detrimental effects of undiagnosed and/or untreated PND include both negative gestational and obstetric outcomes (i.e., preterm delivery, low birth weight, low infant APGAR scores, and developmental delays) as well as increased maternal morbidity and mortality including infanticide, suicidality and self-harm injuries (15–19). Overall, PND risk factors include previous history of pre-conceptional mental illness (particularly MDD), experiences of adverse and/or stressful life events (including adverse childhood events), the lack of social support, low socioeconomic status and unplanned/unwanted pregnancy (3, 20–22).

Indeed, beyond the abovementioned established risk factors for PND, another interesting research and clinical field is represented by the investigation of affective temperamental profiles on the likelihood of developing PND, considering the vast literature already published for all affective spectrum disorders (23–32). Although the concept of temperament in relation to affective disorders has been looked into over the centuries by numerous scholars, including Robert Burton (33) and André de Laurens (34), it was the German psychiatrist Emil Kraeplin who first described the four basic affective dispositions, i.e.: depressive, manic, cyclothymic, irritable (35). Kraeplin developed his temperament theory based on the works of Galen (II century), who formulated the theory of humors – melancholic, choleric, phlegmatic, sanguine – and argued that their imbalance was at the root of human diseases. Kraeplin considered these basic affective temperaments as subclinical forms of manic depressive illness, now known as affective disorders (unipolar, bipolar, schizoaffective), found in patients and also in their families (35). Kraeplin and his German colleague Ernst Kretschmer both believed that affective temperaments could predispose individuals to endogenous psychosis or mood disorders episodes. However, Kretschmer pointed out the concept of dominant/predominant temperament, considering it as a variation of normal affectivity rather than as a necessary condition for the onset of the disorder (36). He proposed that basic temperaments separate two major constitutional groups: cyclothymic versus schizothymic individuals (36). Based on these theories, ranging from ancient humoral concepts to the more modern perspectives of Kraepelin and Kretschmer, and supported by extensive clinical observation and field research, Hagop Akiskal and his collaborators developed the modern concept of affective temperaments (37). Akiskal elaborated his concept of affective temperament by integrating ancient ideas with the most modern scientific observations to encompass the full spectrum of affective disorders, from healthy emotional reactivity to the development of significant affective disorders. His model includes the four classic temperament types along with a fifth component represented by the anxious affective temperament (38). Affective temperament refers to an individual’s innate (genetically determined) inclinations to respond to environmental stimuli with specific and stable feelings, emotions and sensations (39). Five affective temperaments have been proposed constituted by depressive, anxious, irritable, cyclothymic, and hyperthymic (40, 41) (**Table 1**). Previous studies have suggested that temperamental profiles may contribute to mood dysregulation and emotional vulnerability across both clinical and subclinical populations. In particular, cyclothymic and anxious temperaments have been associated with mood instability, affective reactivity, and increased susceptibility to depressive symptoms (48). Moreover, recent findings have highlighted the potential role of affective temperaments and trait-anxiety dimensions in predicting mood alterations and emotional vulnerability, supporting the view that these temperamental traits may represent early vulnerability markers for affective disorders (49–51). These temperamental models of affective psychopathology provide a useful framework for investigating vulnerability factors for perinatal depression. For PND, some studies have suggested that temperamental and personality traits may contribute to vulnerability for postpartum depression. In particular, affective temperaments assessed during pregnancy have been associated with the development of postpartum depressive symptoms (52), and broader personality traits such as neuroticism and trait anxiety have been consistently linked with increased PPD risk (53).

**Table 1:** Affective temperaments features and association with MDD (based on: 37, 40, 42–47).

Affective temperament	Clinical features	Prevalence in clinical sample	Prevalence in general population	Risk of developing depression	Suicidal behavior
Depressive	Pessimistic, skeptical, gloomy, incapable of fun, preoccupied with inadequacy, failure, negative events, given to worry, guilt-prone, low energy, hypersomnia	20.1% F>M	3.4% - 4.7% F>M in women increase with age	The depressive temperament is closely linked to a high likelihood of developing MDD. Clinical studies indicate that over 50% of individuals with depressive temperament may experience depressive episodes in their lifetime	Strongly associated, particularly among females
Hyperthymic	High activity, territoriality, leadership, risk-taking, stimulus-seeking stress-resistance, extroversion, grandiosity, reduced need for sleep	52.7% M>F	0.2% - 3.3% M>F	Generally associated with a low risk of depression, though there is increased risk in transitions to bipolar disorders	Protective factor
Cyclothymic	Social withdrawal alternating with uninhibited sociability, labile self-esteem, overconfidence alternating with low self-confidence, rapid mood fluctuations, emotional instability	15.0% F>M	2.1% - 5.9% F>M	Mood variability and impulsivity increase the risk of depression, especially during transitions to depressive phases. Approximately 30-50% of cyclothymic individuals develop MDD over time	Associated, particularly among females
Irritable	Restless, dysphoric, broody, choleric.	8.04% M>F	2.7% - 6.2% M>F	Lower risk, but it may increase with stressful events or relational traumas	Strongly associated
Anxious	increased orthosympathetic activity, fear of loneliness, hypersensitivity to separation, exaggerated reactions to external stimuli	4.09% F>M	3.1% - 4.6% F>M	The anxious component can lead to secondary depressive episodes, with about 20-30% of anxious individuals developing depression	Not significantly associated

F, females; M, males; MDD, major depressive disorder.

Overall, despite the literature already existing on MDD and associated affective temperamental profiles in illness onset, clinical course, prognosis and treatment response (24–26, 28, 29, 31, 54–57), at the time of present writing, there are few published study investigating the affective temperamental profiles among women with PND. Therefore, the current study aims at exploring the role of affective temperament in women with PND, in order to understand the relationship with the risk of developing PND, including the association with depression severity, in a group of pregnant women recruited in a Perinatal Mental Health outpatient service, involved in screening, assessing women at-risk for PND and pregnant women with a preexisting mental condition in the preconception period. The primary objective was to identify whether a specific affective temperamental profile is predominant among women with PND. The secondary objective was to investigate whether there is a relationship between specific affective temperaments and depressive severity among PND women. The final goal aimed to identify specific temperamental profiles able to clinically characterize pregnant women at-risk for PND, in order to build preventive strategies and personalize clinical interventions accordingly, considering the already available literature clearly demonstrating the association between specific affective temperaments, clinical outcomes (such as illness onset, clinical course, treatment response) and potential detrimental conditions associated with MDD, such as suicidality risk.

## 2 Methods

### 2.1 Study design and selection of participants

The present study is a sub-analysis of a larger multicenter nationwide population-based naturalistic observational screening project aimed at early identifying pregnant and/or puerperal women at-risk for developing perinatal mental disorders. All pregnant women admitted at the Perinatal Mental Health Outpatient Service, Unit of Clinical Psychiatry, University Hospital of Marche, Polytechnic University of Marche, Ancona, Italy, between April 2021 and July 2025, were consecutively recruited and longitudinally screened for perinatal depression (PND). Women were typically referred to this service by obstetricians, gynecologists, primary care physicians, mental health services, or other community healthcare providers when psychological distress or psychiatric vulnerability is identified during pregnancy. In addition, women may also access the service autonomously by directly contacting the outpatient clinic. Written informed consent for research purposes was obtained from all participating women. All women were given the possibility to withdraw their participation from the study, without clinical or therapeutic consequences. Recruitment and enrolment of the final sample were based on the following inclusion criteria: a) ≥ 18 years old; b) pregnant women; c) absence of linguistic and/or learning difficulties (i.e., not fluent Italian speaker and/or without a sufficient capability to understand Italian language); d) signed informed consent for collecting and analyzing clinical data for research purposes. Participants were excluded if they met one or more of the following exclusion criteria: (a) incomplete or inadequately filled out questionnaires; (b) refusal to complete the informed consent; (c) not pregnant women. All the study procedures were in accordance with the ethical standards of the institutional and/or national research committee and with the 1964 Helsinki Declaration and its later amendments or comparable ethical standards. The institutional Ethics Committee approved the study (Prot. 378/2022). This research study was conducted retrospectively from data obtained for clinical purposes.

### 2.2 Measures

All participants were asked to complete a structured case report form including sociodemographic and clinical data (e.g., age, ethnicity, marital status, employment status, education level, family psychiatric history, medical history, pregnancy-related and other health conditions, previous psychiatric disorders and treatment histories). According to the 2018 National Institute for Health and Care Excellence (NICE) guidelines on antenatal and postnatal mental health, all women were administered the Edinburgh Postnatal Depression Scale at the admission to the outpatient unit (58, 59), as a screening tool for detecting depressive symptomatology in pregnancy. For those women positive at the EPDS screening, the clinical diagnosis of antenatal depression was carried out by senior psychiatrists through a semi-structured clinical interview for DSM-5 (SCID-5-CV) (60) administered to all pregnant women, to confirm the PND diagnosis. The EPDS is a self-administered questionnaire consisting of 10 items, commonly utilized in perinatal mental health research (61, 62), and it has been validated for use throughout all stages of the perinatal period (63–65). This scale evaluates the intensity of depressive symptoms experienced by women during the preceding seven days and has been recognized as an effective instrument for the early detection of those at risk of PND (66). Each item is rated on a four-point Likert scale ranging from 0 to 3, resulting in a total score between 0 and 30. The Italian validation studies of the EPDS reported strong internal reliability, with a Cronbach’s α of 0.80 during pregnancy and 0.87 postpartum (67, 68). In our sample, the scale demonstrated excellent internal consistency (Cronbach’s α = 0.936). For the identification of PND, we adopted a cut-off score of ≥12 on the EPDS, in line with prior literature and international recommendations (13, 66, 69, 70). As noted in the validation of the Italian version (59), the optimal cut-off depends on the purpose of the assessment: a threshold of 9 is generally preferred for screening or population-level surveys, whereas a score of 12 is commonly applied in clinical or research settings, particularly when targeting individuals with a higher likelihood of developing PND (13). Furthermore, all participants completed the Italian-validated short form of the Temperament Evaluation of Memphis, Pisa, Paris and San Diego at the admission to the outpatient unit (TEMPS-M; 71), a 35-item questionnaire rated on a 5-point Likert scale, designed to evaluate affective temperaments as conceptualized by Akiskal through a dimensional framework (depressive, anxious, hyperthymic, cyclothymic, and irritable) (37, 42). The TEMPS-M has demonstrated satisfactory internal consistency, with Cronbach’s α values ranging from 0.69 to 0.84 (71), and in our sample, it showed a good reliability (Cronbach’s α = 0.884).

### 2.3 Statistical analysis

Categorical variables (i.e., sociodemographic features, clinical and pregnancy-related variables) were presented by frequencies (n) and percentages (%). Continuous variables, whereas normally distributed, were expressed as mean and standard deviations (SD); whereas not normally distributed, as median and quartiles. The sample was also divided in two groups: those women with PND after a positive screening at EPDS and confirmation through SCID-5-CV (PND+) and those women without PND (PND-). The prevalent temperament was obtained in each patient considering the highest score obtained at TEMPS-M subscales (71). Given the dimensional nature of affective temperaments, this approach was used as a descriptive method to characterize the predominant temperament profile, acknowledging that individuals may present elevated scores across multiple temperament dimensions. The Mann-Whitney U test was also used to compare the mean scores of the TEMPS-M subscales obtained by PND-/PND+ women and the mean EPDS total score in categorical variables (presence or absence of previous psychiatric illness, family history of psychiatric illness, and presence or absence of medical comorbidities). To compare prevalent temperament (as categorical variable) among PND+/PND- groups, we used the χ2 Test. A stepwise multivariate linear regression was used to assess the relationship between EPDS total score (as depressive severity measure) and each TEMPS-M subscales. Furthermore, a stepwise binary logistic regression analysis was performed in order to evaluate the TEMPS-M subscales associated with the presence of PND (*vs*. the absence of PND). The estimated odds ratios (OR) along with the 95% of confidence intervals (95% CI), and standardized coefficient β values were generated for each variable. For both regression analyses, potential confounding variables (age, positive psychiatric history, presence of medical comorbidities, and family psychiatric history) were initially included in the regression models but were not retained in the final stepwise solution. Multicollinearity among predictors was assessed using Variance Inflation Factor (VIF). No evidence of problematic multicollinearity was detected (VIF < 5). For all analyses, the level of statistical significance was set at p < 0.05, two-tailed. Statistical analysis was performed using SPSS version 25 for Windows.

## 3 Results

### 3.1 Socio-demographic, clinical and psychopathological characteristics of the participants

All socio-demographic characteristics of the study participants are summarized in **Table 2**.

**Table 2:** Socio-demographic characteristics of the sample.

Variables	Total sample		PND-		PND+	
	N	%	N	%	N	%
Ethnicity						
Caucasian	122	91.7%	84	94.4%	38	86.4%
Asian	3	2.3%	1	1.1%	2	4.5%
African	3	2.3%	2	2.2%	1	2.3%
Other	5	3.8%	2	2.2%	3	6.8%
Illness onset						
Ante-Partum	105	78.9%	74	83.1%	31	70.5%
Post-Partum	28	21.1%	15	16.9%	13	29.5%
Educational level						
Elementary school degree	2	1.5%	0	0%	2	4.5%
Middle school diploma	10	7.5%	6	6.7%	4	9.1%
High school diploma	48	36.1%	29	32.6%	19	43.2%
Degree or higher	73	54.9%	54	60.7%	19	43.2%
Marital status						
Single	7	5.3%	3	3.4%	4	9.1%
Married or in a stable relationship	125	94.0%	86	96.6%	39	88.6%
Divorced or Separated	1	0.8%	0	0%	1	2.3%
Working status						
Unemployed	36	27.1%	19	21.3%	17	38.6%
Employed	97	72.9%	70	78.7%	27	61.4%
Smoking habits						
No	90	67.7%	58	65.2%	32	72.7%
Yes	37	27.8%	30	33.7%	7	15.9%
Previous	6	4.5%	1	1.1%	5	11.4%
Substance use						
No	118	88.7%	81	91.0%	37	84.1%
Yes	9	6.8%	7	7.9%	2	5%
Previous	6	4.5%	1	1.1%	5	11.4%
Positive psychiatric history						
No	64	48.1%	55	61.8%	9	20.5%
Yes	69	51.9%	34	38.2%	35	79.5%
Family history of mental disorders						
No	67	50.4%	54	60.7%	13	29.5%
Yes	66	49.6%	35	39.3%	31	70.5%
Previous pregnancy						
No	83	62.4%	57	64%	26	59.1%
Yes	50	37.6%	32	36%	18	40.9%
Voluntary interruption of pregnancy (VIP)						
No	120	90.2%	83	93.3%	37	84.1%
Yes	13	9.8%	6	6.7%	7	15.9%
Previous spontaneous abortion						
No	117	90.2%	83	93.3%	34	77.3%
Yes	16	9.8%	6	6.7%	10	22.7%

Among all patients consecutively evaluated in the timeframe 2021, April - 2025, July (N = 180) who agreed to participate to the study, after excluding those not pregnant and/or not puerperal women (N = 11) and those with incomplete questionnaires (N = 36), a final sample of 133 were definitely recruited in the study. The mean age was 33.9 years (SD = 5.9). Most participants were Caucasian (91.7%; N = 122), married or in a stable relationship (94%; N = 125), had a university degree (54.9%; N = 73) and declared to be regularly employed (72.9%; N = 97)(**Table 2**).

The mean weight before pregnancy was 62 Kg (SD = 6.1) with a mean BMI of 22.9 Kg/m^2^ (SD = 4.8). Most of the sample had an antepartum onset of affective disorders (74.4%; N = 99). Around 27.8% of the sample (N = 37) reported to be active smokers, while 6.8% (N = 9) of them declared a current substance use. Around 49.6% (N = 66) of participants had a positive family history for psychiatric disorders and 51.9% (N = 69) of them had a previous psychiatric history (**Table 3**). Those with a history of psychiatric disorders (p<0.001) and those with a family history of psychiatric disorders (p = 0.003) obtained the highest EPDS scores. Around 37.6% (N = 50) had a previous pregnancy, 9.8% (N = 13) had a voluntary interruption of pregnancy (VIP), 12% (N = 16) had a previous spontaneous abortion. Almost half of the sample had a significant medical comorbidity (n = 78; 58.6%).

**Table 3:** Comparison of the mean scores obtained in the EPDS total score across all categorical variables.

Variables	Mean	Standard deviation	P-value
Ethnicity			
Caucasian	8.9	7.7	0.8
Asian	14.0	12.2	
African	9.7	9.1	
Other	12.4	8.8	
Illness onset			
Ante-Partum	8.3	7.5	**0.012**
Post-Partum	12.5	8.1	
Educational level			
Elementary school degree	20.0	2.8	0.359
Middle school diploma	9.5	10.4	
High school diploma	9.5	8.3	
Degree or higher	8.7	7.0	
Marital status			
Single	15.0	10.7	0.166
Married or in a stable relationship	8.8	7.5	
Divorced or Separated	17.0	/	
Working status			
Unemployed	11.1	8.9	0.259
Employed	8.5	7.3	
Smoking habits			
No	9.3	7.9	**0.046**
Yes	7.7	7.1	
Previous	16.2	6.8	
Substance use			
No	8.7	7.4	**0.004**
Yes	7.7	7.6	
Previous	20.7	6.8	
Positive psychiatric history			
No	5.6	5.8	**<0.001**
Yes	12.5	7.9	
Family history of mental disorders			
No	7.1	6.8	**0.003**
Yes	11.3	8.2	
Previous pregnancy			
No	8.8	7.8	0.342
Yes	9.9	7.7	
Voluntary interruption of pregnancy (VIP)			
No	8.7	7.4	0.092
Yes	13.5	9.7	
Previous spontaneous abortion			
No	8.6	7.6	**0.013**
Yes	13.7	7.7	
Medical comorbidities			
No	10.4	8.0	0.160
Yes	8.3	7.6	

In bold significant p-values.

### 3.2 Clinical and psychopathological predictors of perinatal depression

The mean EPDS total score was 9.2 (SD = 7.8), with a prevalence rate of 33.1% of PND, according to the established cut-off of 12. **Table 4** describes mean scores of each TEMPS-M subscales of the sample. The prevalent temperament of the sample was the hyperthymic (56.4%; N = 75) and the depressive (18%; N = 24).

**Table 4:** Mean scores obtained in the TEMPS-M subscales by the sample and comparison between PND-/PND+ subjects.

Variables	Total sample		PND-		PND+		P-value
	Mean	SD	Mean	SD	Mean	SD	
TEMPS-M depressive subscale	17.1	8.3	15.1	8.2	21.2	6.8	**<0.001**
TEMPS-M cyclothymic subscale	14.0	6.3	11.0	5.5	17.7	6.2	**<0.001**
TEMPS-M hyperthymic subscale	20.7	5.3	21.5	5.1	19.0	5.4	**0.006**
TEMPS-M irritable subscale	13.0	4.7	12.5	3.9	14.1	5.7	0.198
TEMPS-M anxious subscale	15.9	5.9	14.3	5.2	19.0	5.9	**0.001**

SD, standard deviation; TEMPS-M, Temperament Evaluation of Memphis, Pisa, Paris and San Diego. In bold significant p-values.

**Figure 1:**
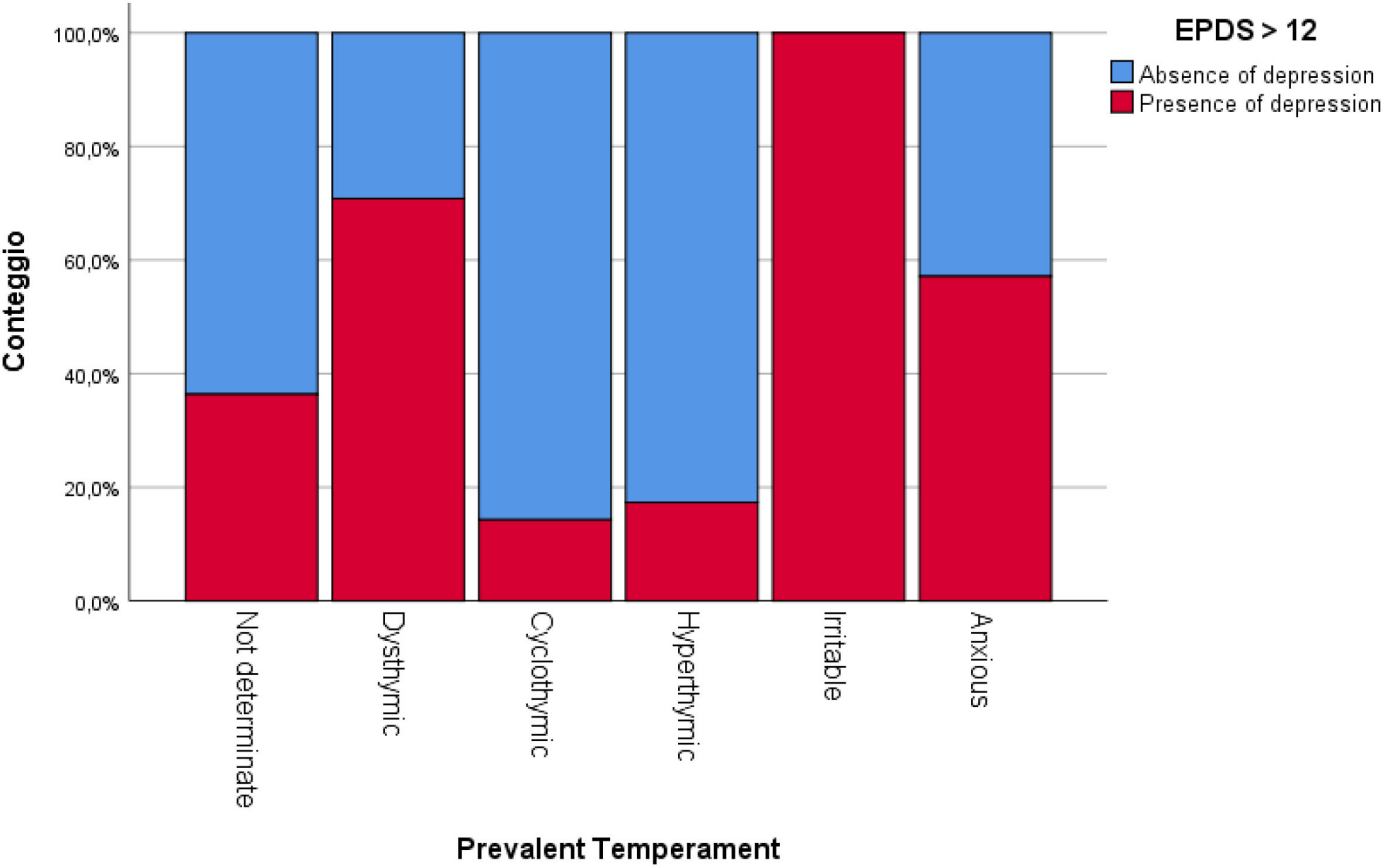
Prevalence of PND (according to EPDS total score) based on predominant affective temperament.

Patients with predominant depressive, irritable and anxious affective temperaments displayed the highest prevalence of PND (p<0.001) (**Figure 1**).

According to the multivariate linear regression model, higher scores at the EPDS were positively associated with TEMPS-M cyclothymic subscale (B = 0.356, p = 0.001), TEMPS-M anxious subscale (B = 0.247, p = 0.026) and a positive personal psychiatric history (B = 5.245, p < 0.001) (R = 0.6, R^2^ = 0.36, F(3,129) = 24.189, p < 0.001) (**Table 5**).

**Table 5:** Multivariate linear regression model with EPDS total score as dependent variable.

Variables	B	SE	β	t	P-value	95% CI Lower Limit	95% CI Upper Limit
TEMPS-M cyclothymic subscale	0.356	0.101	0.288	3.541	**0.001**	0.157	0.555
Positive psychiatric history	5.245	1.142	0.338	4.593	**<0.001**	2.986	7.505
TEMPS-M anxious subscale	0.247	0.110	0.186	2.245	**0.026**	0.029	0.464

SE, Standard Error; CI, Confidence Interval; TEMPS-M, Temperament Evaluation of Memphis, Pisa, Paris and San Diego. In bold significant p-values.

A logistic regression analysis was performed to ascertain the effects of all types of five affective temperaments (as measured by TEMPS-M) on the likelihood of developing a PND (with a EPDS total score higher than 12), adjusting for confounding factors. The logistic regression model was statistically significant, χ2(5) = 55.395, p < 0.001. The model explained 47.4% (Nagelkerke R2) of the variance in peripartum depression and correctly classified 77.4% of cases. According to the logistic regression model, PND was significantly associated with higher scores at TEMPS-M cyclothymic and anxious subscales, lower score at TEMPS-M hyperthymic subscale, a positive psychiatric history and the absence of other medical comorbidities (**Table 6**).

**Table 6:** Binary logistic regression analysis predicting the presence of PND (as dependent variable, according to EPDS total score).

Variables	B	SE	Wald	df	P-value	Exp (B)	95% CI Exp (B)	
Presence of medical comorbidities	-1.494	0.505	8.749	1	**0.003**	0.224	0.083	0.604
Positive psychiatric history	1.638	0.508	10.377	1	**0.001**	5.144	1.899	13.935
TEMPS-M cyclothymic subscale	0.112	0.042	7.134	1	**0.008**	1.118	1.030	1.214
TEMPS-M hyperthymic subscale	-0.094	0.048	3.892	1	**0.049**	0.911	0.830	0.999
TEMPS-M anxious subscale	0.103	0.047	4.795	1	**0.029**	1.109	1.109	1.217

SE, Standard Error; CI, Confidence Interval; TEMPS-M, Temperament Evaluation of Memphis, Pisa, Paris and San Diego; PND: perinatal depression. In bold significant p-values.

## 4 Discussion

To the best of our knowledge, this is one of the first Italian studies specifically exploring the role of affective temperamental profile in a real-world clinical group of pregnant women with PND recruited in a specialized Perinatal Mental Health outpatient service, by using TEMPS-M. Our study found a PND prevalence rate of 33.1%. With respect to the prevalence, the proportion of women meeting criteria for PND in our sample was higher compared to the range already reported for Italian populations, whereas similar EPDS cut-offs are used (12, 13, 29, 72–74). In fact, large multicenter data and a recent national meta-analysis indicate for PND, antenatal risks around 6%–20% and postpartum risks around 11%–20% with EPDS ≥12 in community samples (12, 13, 29, 72–74). Indeed, our sample is mainly constituted by those highly selected women, already positively screened for PND at peripheral and/or hospital gynecological outpatient services as they were enrolled only when they arrived at our super specialized outpatient perinatal psychopathology service. Therefore, being a not general sample, one could argue that the high PND rate was due to the clinical sample. Overall, our sample is balanced enough in terms of pregnant women reporting a previous positive psychiatric history and/or a family psychiatric history, by allowing to compare both groups depending on these variables.

In line with the primary objective of our study, we found that cyclothymic and anxious affective temperaments and lower hyperthymic affective temperament, were independently associated with PND, according to both multivariate linear regression and binary logistic regression models. Affective temperaments and trait-anxiety dimensions are generally considered relatively stable personality traits that may reflect enduring vulnerability to affective dysregulation. Within this perspective, cyclothymic and anxious temperaments may represent longitudinal vulnerability markers that contribute to the emergence of mood symptoms across different phases of the perinatal period. These findings on affective temperamental profiles in women with PND align with evidence that stable vulnerability traits are central to perinatal psychopathology. In Italian pregnant samples, depressive, cyclothymic and anxious temperaments have repeatedly emerged as risk dimensions, whereas hyperthymic traits appear relatively protective, mirroring patterns observed for general neurotic, emotionally unstable personality profiles in predicting higher perinatal depressive symptomatology and poorer resilience (75–78). Studies using TEMPS-A in Italian pregnancy cohorts show that women with current depressive symptoms score higher on depressive and other non-hyperthymic temperaments, and that these traits independently predict antenatal depressive symptomatology (75). Network analyses further indicate that cyclothymic, depressive and anxious temperaments are the temperamental nodes most tightly linked to PND and generalized anxiety across trimesters, with hyperthymic temperament remaining peripheral (77).

Following the secondary objective of the present study, higher depressive severity, as assessed by EPDS, was positively associated with higher scores on the cyclothymic and anxious TEMPS-M subscales, and a positive personal psychiatric history, suggesting that a predominant cyclothymic-anxious affective temperamental profile as well as personal psychiatric history may represent significant risky factors for PND and PND severity. Previous studies have reported associations between anxious and cyclothymic temperaments assessed in pregnancy and postpartum depressive symptoms (79). Our finding that a cyclothymic affective temperament predominates among women with PND is consistent with previous evidence linking cyclothymic traits to greater affective instability, more severe depressive episodes, and a more complex clinical course (such as higher risk of psychotic symptomatology, suicidality, melancholic/atypical symptoms) (24, 37, 40, 42–47). Studies in non-perinatal populations have shown that cyclothymic temperament is associated with earlier onset of mood symptoms, higher risk of recurrence, and a greater likelihood of mixed or atypical features, which in turn may worsen prognosis and functional outcomes. Moreover, cyclothymic temperament has been related to poorer or heterogeneous response to standard antidepressant treatments and an increased need for mood-stabilizing strategies, suggesting that it may serve as a clinical marker of “soft” bipolarity within depressive spectra. Similarly, anxious temperament has been already described to be over-represented among women with perinatal depressive symptomatology compared with non-depressed controls (75). Affective-temperament studies in broader mood-disorder groups further indicate that anxious temperament is linked to greater illness severity and reduced antidepressant response, whereas hyperthymic temperament is associated with more favorable outcomes (56, 80).

Despite the interesting findings of the present study, several limitations must be considered. The main limitation of this study is its retrospective and cross-sectional design, which does not allow causal conclusions either identifying the directionality of the associations found hereby. Secondly, another important limitation is the sampling strategy (risk of selection bias), derived by the fact that findings were collected by a monocentric, second-level specialized center specifically addressed to clinical samples and not a community random sample. This would explain the high prevalence of PND in the sample. Therefore, the results carried out by recruiting convenience samples can only be generalized to the conveniently accessible population from which the sample was drawn. Thirdly, our study administered a self-report tool to assess affective temperament (i.e., TEMPS-M), possibly affecting the responses due to social desirability and common method biases. At the same time, state variables may have influenced the patients’ response to the scale. Future studies should be carried out by using longitudinal design and predictive statistical modeling to understand the temporal dynamics and causal relationships between the clinical outcomes of PND (clinical course, treatment response, and suicidality risk) and these specific affective temperamental profiles (particularly, cyclothymic and anxious). Moreover, further studies should also consider the enrollment of both pregnant and puerperal women (by comparing findings according to the affective temperamental profile) as well as compare clinical samples of women affected with MDD with versus without a positive psychiatry history of PND.

Therefore, consistent with published literature, our results reinforce the view that PND in Italy is common and strongly shaped by enduring affective dispositions, suggesting that routine assessment of affective temperament—alongside socioeconomic and interpersonal risk factors—may help identify women at particularly high risk within this already vulnerable group (12, 13, 72, 75, 76, 78, 81). Within the perinatal context, our results extend this literature by indicating that predominant cyclothymic and anxious affective temperaments may help identify a subgroup of women with PND who are more likely at heightened risk for mood instability, greater anxiety/mixed symptomatology in comorbidity, and suboptimal treatment response, thereby underscoring the clinical value of systematic temperamental assessment to inform individualized prevention and treatment plans, as well as to prognosis, intensity of monitoring, including for suicidality risk. These findings are in line with the final goal of this real-world exploratory study, by suggesting further research developments including prospective longitudinal cohort studies to assess the effective impact of this predominant affective temperamental profile on clinical outcomes (such as illness onset, clinical course, treatment response, suicidality risk) among pregnant and puerperal women.

## Data Availability

The data that support the findings of this study are available from the corresponding author upon reasonable request. The data are not publicly available due to ethical restrictions and the need to protect the privacy of research participants.
